# Characterization of Genome-Wide Association-Identified Variants for Atrial Fibrillation in African Americans

**DOI:** 10.1371/journal.pone.0032338

**Published:** 2012-02-23

**Authors:** Jessica T. Delaney, Janina M. Jeff, Nancy J. Brown, Mias Pretorius, Henry E. Okafor, Dawood Darbar, Dan M. Roden, Dana C. Crawford

**Affiliations:** 1 Department of Medicine, Vanderbilt University, Nashville, Tennessee, United States of America; 2 Department of Molecular Physiology and Biophysics, Vanderbilt University, Nashville, Tennessee, United States of America; 3 Center for Human Genetics Research, Vanderbilt University, Nashville, Tennessee, United States of America; 4 Department of Pharmacology, Vanderbilt University, Nashville, Tennessee, United States of America; 5 Department of Anesthesiology, Vanderbilt University, Nashville, Tennessee, United States of America; 6 Department of Medicine, Meharry Medical College, Nashville, Tennessee, United States of America; Ohio State University Medical Center, United States of America

## Abstract

**Background:**

Despite a greater burden of risk factors, atrial fibrillation (AF) is less common among African Americans than European-descent populations. Genome-wide association studies (GWAS) for AF in European-descent populations have identified three predominant genomic regions associated with increased risk (1q21, 4q25, and 16q22). The contribution of these loci to AF risk in African American is unknown.

**Methodology/Principal Findings:**

We studied 73 African Americans with AF from the Vanderbilt-Meharry AF registry and 71 African American controls, with no history of AF including after cardiac surgery. Tests of association were performed for 148 SNPs across the three regions associated with AF, and 22 SNPs were significantly associated with AF (P<0.05). The SNPs with the strongest associations in African Americans were both different from the index SNPs identified in European-descent populations and independent from the index European-descent population SNPs (r^2^<0.40 in HapMap CEU): 1q21 rs4845396 (odds ratio [OR] 0.30, 95% confidence interval [CI] 0.13–0.67, P = 0.003), 4q25 rs4631108 (OR 3.43, 95% CI 1.59–7.42, P = 0.002), and 16q22 rs16971547 (OR 8.1, 95% CI 1.46–45.4, P = 0.016). Estimates of European ancestry were similar among cases (23.6%) and controls (23.8%). Accordingly, the probability of having two copies of the European derived chromosomes at each region did not differ between cases and controls.

**Conclusions/Significance:**

Variable European admixture at known AF loci does not explain decreased AF susceptibility in African Americans. These data support the role of 1q21, 4q25, and 16q22 variants in AF risk for African Americans, although the index SNPs differ from those identified in European-descent populations.

## Introduction

Atrial fibrillation (AF) is the most frequent arrhythmia in clinical practice, with over 2.3 million Americans diagnosed with the disease [1]). The incidence of AF varies by race, occurring more frequently in European Americans than in African Americans [2], [3]. Paradoxically, risk factors for AF including hypertension (HTN), obesity, diabetes mellitus (DM), and congestive heart failure (CHF) are more prevalent in African Americans [3]–[7]. As a result, traditional risk factors used to assess AF risk among African Americans perform poorly in this population.

Over the last few years, several genetic variants have been associated with AF risk in genome-wide association studies (GWAS) performed in populations of European-descent [8]. To date, three genomic regions/candidate genes have been identified: 4q25 near *PITX2*, in *ZFHX3* on 16q22, and in *KCNN3* on 1q21 [9]–[12]. For the 4q25 locus, rs2200733 has been consistently the SNP with the highest risk in multiple GWAS cohorts and in a fine mapping study of the region [13]. The 4q25 locus also includes other SNPs that predict risk and are not in linkage disequilibrium with rs2200733 [13]. For both *ZFHX3* and *KCNN3*, the reported index SNP has varied among studies likely reflecting the strong linkage disequilibrium observed for these genomic regions in European-descent populations [9]–[14]. As no GWAS has yet been performed for AF in populations of African-descent, it remains to be determined whether there is an association with these regions and if there is, whether the index European variants from these three genomic regions generalize to African Americans or if there are additional variants in the same genomic regions more strongly associated with AF in populations of non-European-descent.

African-descent populations such as African Americans are often used for fine-mapping experiments based on the different allelic architecture and lower levels of linkage disequilibrium of these populations compared with European Americans [15]. African Americans, however, are also a genetically heterogeneous population, with varying European and African contributions. It is possible that the proportion of European ancestry (EA) can modify the risk of AF [16]–[18]. Indeed, genetic ancestry has been associated with disease risk for many diseases including obesity, hypertension (HTN), diabetes mellitus (DM), kidney disease, asthma, rheumatoid arthritis, breast cancer, and prostate cancer [19]–[29]. Recently, the proportion of European ancestry in African Americans was associated with the development of AF in individuals enrolled in the Cardiovascular Health Study (CHS) and Atherosclerosis Risk in Communities (ARIC) Study [30].

We report here a targeted genetic association study of AF in African Americans ascertained at Vanderbilt University in Nashville, TN. We first sought to generalize in African Americans three regions associated with AF risk in European-descent populations. We then calculated estimates of genetic ancestry and tested for associations with AF in African Americans. We demonstrate that for the three regions discovered in European-descent populations do generalize with respect to direction of effect, although the best index genetic variant associated with AF in African Americans is different. Our data also suggest that in contrast to previous reports based on genome-wide data, AF is not associated with admixture in this dataset for these three genomic regions.

## Methods

### Ethics Statement

The study protocol was approved by the Vanderbilt University Institutional Review Board. A written informed consent was obtained from all patients prior to enrollment.

### Study Population

Cases were selected from patients consecutively enrolled in the Vanderbilt-Meharry AF registry who self-reported African American race. Details of the Vanderbilt-Meharry AF registry have been previously published [31]. In brief, from 2002 to present, the registry has been prospectively enrolling patients with newly diagnosed AF or a documented history of AF, excluding those who developed atrial fibrillation in the post-operative period after cardiac surgery. Clinical data and blood samples were obtained at the time of enrollment.

Controls were selected from the Vanderbilt Cardiac Surgery registry, a registry of consecutive patients undergoing cardiac surgery [32]. Controls were selected among those who did not develop AF in the post-operative period from cardiac surgery and self-reported African American ancestry. Patients with a personal or family history of AF were excluded. The study protocol was approved by the Vanderbilt University Institutional Review Board, and participants were enrolled after informed consent was obtained.

### SNP selection

We selected SNPs based on three index associations observed in European-descent populations for AF. The three index SNPs include rs2200733, an intergenic SNP located at 4q25 near *PITX2*; rs13376333, an intronic SNP located at 1q21 in *KCNN3*; and rs2106261 an intronic SNP located at 16q22 in *ZFX* [9]–[12]. Regions of interest from the index SNP were identified in African American samples from the southwest (ASW) in the International HapMap Project using the Genome Variation Server (http://gvs.gs.washington.edu/GVS/). We implemented LDSelect to identify tagSNPs in the three regions of interest for genotyping [33]. In brief, all SNPs in the region were filtered by minor allele frequency (MAF 5%) in the ASW sample. Common SNPs were then binned by linkage disequilibrium (r^2^) for each of the three regions based on the ASW data. A tagSNP was selected from each bin with the criteria that the tagSNP was in strong LD with the other SNPs in the bin (r^2^ threshold of 0.80) but independent (i.e. not in strong LD) with other tagSNPs across bins. If multiple tagSNPs were available per bin, priority was given to the tagSNP identified in prior European descent AF GWAS studies followed by those with the highest PhastCons Conservation Score. A total of 51 tagSNPs around rs1337633, 50 tagSNPs around rs2200733, and 47 tagSNPs around rs2106261 were included for genotyping. Due to varying linkage disequilibrium, the genomic distance covered by selected tagSNPs varied: 400 kb for rs2200733, 200 kb for rs13376333, and 160 kb for rs2106261.

### SNP Genotyping

Genotyping was performed by the Vanderbilt DNA Resources Core with either Sequenom iPLEX Gold® assay on the MassARRAY® Platform or Applied Biosystem's TaqMan. All SNP call rates were >95%, with an average call rate of 99%. Three samples were excluded from the analysis due to low genotyping efficiency (<95%). Tests of Hardy-Weinberg equilibrium (HWE) were calculated separately for cases and controls, and no SNP deviated from HWE (p<0.0003).

### Statistical Methods

Differences between cases and controls were identified using a chi-square test or a Student's t-test for variables listed in Table 1. Using logistic regression implemented in PLINK [34], tests of association were performed both unadjusted and adjusted for age, body mass index (BMI), prevalent congestive heart failure (CHF), prevalent diabetes mellitus (DM), prevalent HTN, and prevalent coronary artery disease (CAD) assuming an additive genetic model for all SNPs that passed quality control described above (148 SNPs). All adjusted tests of association are shown in Table S1. Comparison of the allele frequencies for the ten most significantly associated SNPs of cases and controls to HapMap Phase II CEU and West African (YRI) samples was performed with chi-square test. The fixation index F_ST_, a measure of population differentiation, was calculated using the Weir and Cockerham algorithm [35] implemented in the Platform for the Analysis, Translation, and Organization of large-scale data (PLATO) [36]. Correction for multiple comparisons by permutation testing and calculation of the False Discovery Rate (FDR) was performed in (PLINK) [34].

**Table 1:** Study Population Characteristics.

Trait	Case (n = 73)	Control (n = 71)	p-value
Age, y	50.2±1.7	53.7±1.5	0.06
Gender, %female	42.4	56.3	0.09
Body Mass Index, kg/m^2^	35.1±1.1	28.8±0.8	<0.001
Left Atrial Size	44.4±1.5	42.4±1.3	0.58
Left Ventricular Ejection Fraction, %	46.4±2.1	46.3±1.7	0.19
Hypertension, %	84.9	84.5	0.94
Diabetes, %	26	35.2	0.23
Congestive heart failure, %	40.9	25.4	0.05
Stroke, %	19.1	17.9	0.89
Coronary artery disease, %	26.8	71.4	<0.001
Current smoker, %	19.4	31.4	0.11
% European Ancestry	23.6	23.8	0.64

Mean (± standard errors) or proportions are given for each variable among cases and controls. A chi-square test or a Student's t-test was performed to test for differences between cases and controls, where appropriate.

Using the risk estimate of AF reported in Europeans (OR = 1.2), percent European ancestry within the three loci was calculated and averaged over all independent SNPs (r^2^<0.8) using ANCESTRYMAP [37]. In addition, local estimates of European ancestry were calculated using HAPMIX [38]. This method determines the likelihood that a haplotype from an admixed individual is derived from one reference population using phased haplotype information from the International HapMap Project. For our African American samples, we used phased haplotype data from CEU and YRI HapMap Phase II. We calculated the probability for each individual having 0, 1, or 2 copies of a European allele at each locus. For each individual, we averaged the number of copies of European alleles over all SNPs for each chromosome separately. Logistic regressions with AF case-control status as the dependent variable and estimates of European ancestry as the independent variable were performed. Logistic regression with adjustment for ancestry in addition to the covariates discussed above was also performed for all SNPs.

Using Quanto we calculated our power to detect GWAS-identified associations in our African American study population [39], [40]. We assumed the reported effect size (OR) in Europeans to calculate the power to detect this effect given our sample size and minor allele frequencies in African Americans.

## Results

### Study Population

Of the 1,624 patients enrolled in the Vanderbilt-Meharry AF registry, 73 were self-identified as African American. From the Vanderbilt Cardiac Surgery registry, 71 controls were identified for comparison. Cases and controls did not differ with respect to mean age or proportion of females (Table 1). Cases had a higher mean body mass index (BMI; 35.1 kg/m^2^) and higher prevalence of CHF (40.9%) compared with controls (28.8 kg/m^2^ and 25.4%, respectively, p<0.001; Table 1). Controls had an increased prevalence of CAD compared with cases (71.4% versus 26.8%, p<0.001), which is consistent with their selection from a post-operative cardiac surgery registry.

### Association results

A total of 22/148 SNPs were associated with AF in adjusted models at P<0.05 (Table 2). Further adjustment for European ancestry did not appreciably alter the results (data not shown). The greatest proportion of significant associations among tested SNPs was observed in the region of 4q25 (15/50; 30%), followed by 1q21 (6/51; 11.8%), and 16q22 (1/47; 2.1%). The most significant SNP in each region in this African American dataset differed from the index SNPs from previously published European-descent populations: rs4631108 (4q25), rs16971547 (1q21), and rs4845396 (16q22) (Figures S1,S2,S3). Similar to the index SNPs identified in European Americans, the African American index SNPs were either intergenic (rs4631108) or intronic (rs16971547 and rs4845396).

**Table 2:** SNPs associated with atrial fibrillation in African Americans.

SNP	CHR	Chromosomal Location	Coded Allele	OR	95% Confidence Interval	P-value
rs4631108	4	111773867	A	3.430	1.587–7.417	0.002
rs4845396	1	154828409	A	0.298	0.134–0.663	0.003
rs2200733	4	111929618	T	3.283	1.495–7.207	0.003
rs1906602	4	111713323	C	5.926	1.795–19.570	0.004
rs4845397	1	154832304	C	0.321	0.147–0.699	0.004
rs2634071	4	111669220	A	2.810	1.353–5.836	0.006
rs4605724	4	111685081	A	4.723	1.535–14.530	0.007
rs2723334	4	111688752	G	0.376	0.183–0.774	0.008
rs6843082	4	111718067	G	2.629	1.284–5.383	0.008
rs12647316	4	111649251	T	3.760	1.399–10.100	0.009
rs10516563	4	111677722	G	3.283	1.332–8.097	0.010
rs11264275	1	154825270	G	0.385	0.181–0.820	0.013
rs4277843	4	111777749	G	2.280	1.173–4.434	0.015
rs6426987	1	154815257	C	0.454	0.240–0.861	0.016
rs16971547	16	73075708	C	8.162	1.467–45.420	0.016
rs6838973	4	111765495	T	0.365	0.157–0.852	0.019
rs13376333	1	154814353	T	2.292	1.124–4.672	0.022
rs4285153	4	111778733	A	0.506	0.265–0.966	0.039
rs11098092	4	111798201	A	2.143	1.037–4.432	0.040
rs3866823	4	111782436	T	2.281	1.033–5.040	0.041
rs11930528	4	111660194	T	2.103	1.025–4.315	0.043
rs1984285	1	154796895	G	0.287	0.083–0.988	0.048

SNP, location (NCBI.36), coded allele, odds ratios, 95% confidence intervals, and p-value are shown for significant associations (p<0.05) after adjusting for age, BMI, history of CAD, history of CHF, history of diabetes, and history of hypertension.

**Figure 1:**
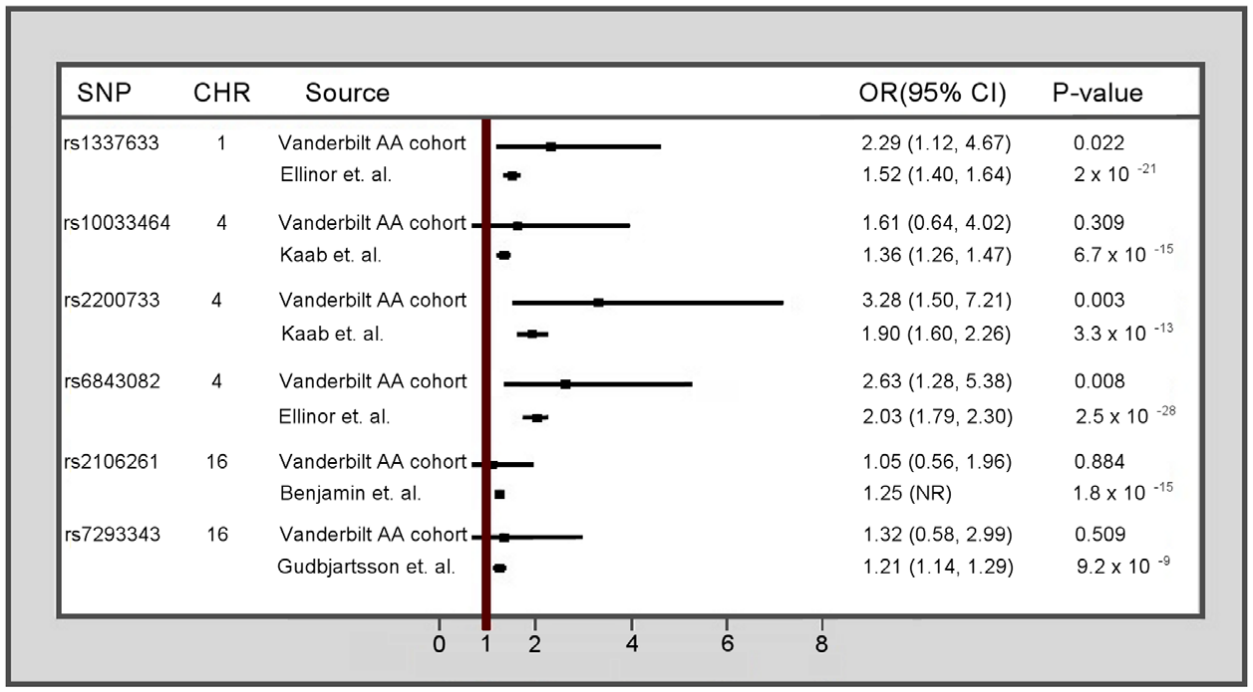
A comparison of previously identified significant SNPs from literature to African Americans from Vanderbilt AF cohort. The case/control sample size for the previously published studies are: Ellinor et. al n = 14,179 (11); Kaab et. al. n = 36,196 (9); Benjamin et. al. n = 46,736 (10); and Gudbjartsson et al. n = 39,014 (50). AA =  Subjects with self-reported African American race.

Of the three European-descent index SNPs, both rs2200733 and rs1337633 were significantly associated with AF among African Americans, while rs2106261 was not. Regardless of significance, the direction of effect for all three index SNPs in African Americans was consistent with that previously reported for European-descent populations (Figure 1). In addition to the index SNPs, three additional SNPs also previously reported to be strongly associated with AF within the selected region were evaluated: rs10033464, rs6843082, and rs7193343. Only rs6843082 was significantly associated in this African American sample. Again, regardless of significance, all three of these SNPs maintained a consistent direction of effect compared with European-descent populations (Figure 1).

### Genetic Ancestry

Given that African Americans have a greater risk for AF but the incidence is lower compared with European Americans, we tested the hypothesis that genomic regions associated with AF in African Americans would have a higher percentage of European ancestry compared with genome-wide averages [2], [3]. This was tested using two strategies. First, we calculated F_ST_ between CEU and case samples, and we compared minor allele frequencies in cases and controls to HapMap CEU and YRI samples for the ten most significant SNPs (Table S2). Case allele frequencies differed from CEU allele frequencies for all ten SNPs (100%), while only 40% (4/10) differed in comparison to YRI. For the controls, 50% (5/10) of SNPs had different allele frequencies when compared to CEU, while 60% (6/10) differed when compared to YRI. F_ST_ estimates did not reveal major differences between cases and CEU reference samples (Table S2).

Second, we estimated admixture in cases and controls using ANCESTRYMAP and HAPMIX, respectively, and tested these estimates for an association with AF. Estimates of admixture from ANCESTRYMAP did not differ between cases and controls (23.6% and 23.8%, respectively; Table 1), and thus did not predict case-control status in this dataset. Local estimates of European ancestry for all three regions were calculated using HAPMIX. For chromosome 1, on average, only 2% of cases had two copies of the European derived chromosome in this region while no controls had two copies. On chromosome 4, 50% of cases had two copies of the European chromosomes at this region compared to 53% of controls. On chromosome 16, ∼2% of cases and controls had two copies of the European derived chromosome. Based on two estimates of admixture, cases and controls did not differ in proportion of European ancestry at any of the loci tested.

## Discussion

We demonstrate here that the European index SNPs from three genomics regions associated with AF are not the index SNPs associated with AF in African Americans. Using two estimates of admixture, we also show that European admixture at any of the genomic regions tested is not associated with increased AF risk in this African American cohort. Our results suggest that AF remains a complex genetic disease as the discordant findings of lower disease prevalence in African Americans, despite the greater incidence of AF clinical risk factors, cannot be explained by European admixture within the 1q21, 4q25, and 16q22 regions.

Of the six SNPs previously reported to be strongly associated with AF in GWA studies of European-descent populations, only three were found to be associated in African Americans, and their effect was reduced in comparison to other SNPS within the regions of interest. SNP rs2200733 in the 4q25 region has consistently replicated in European American populations as the index SNP, whereas in our African American cohort, rs4631108 was found to have the strongest association. A recent fine mapping study of the 4q25 region in European-descent populations identified two additional tagSNPs with a strong association with AF [13]. Of these two SNPs, one (rs3853445) was included in our SNP selection. In adjusted analyses, this SNP was not associated with AF in African Americans (P = 0.164; Table S1). Similar to the 4q25 region, the index SNP observed in African Americans differed from that reported in European-descent populations for both 1q21 and 16q22 genomic regions.

The SNPs included in this study are located in intronic and intergenic regions, both of which are often thought of as regions without obvious function. However, variants in these regions may modify regulatory elements, ultimately resulting in disturbed protein expression. The nearest gene target for the intergenic SNPs found in the 4q25 region is *PITX2*, a gene believed to be involved in cardiac development, specifically sinus node development and susceptibility to atrial arrhythmias [41], [42]. The 1q21 loci is intronic to *KCNN3*, a gene encoding a voltage-independent calcium-activated potassium channel, which has been found in a rabbit model to modify the action potential duration of atrial tissue resulting in increased AF susceptibility, yet, the contribution of this channel to repolarization in humans is unknown [43]. The SNPs located at 16q22 are intronic for *ZFHX3*, a transcription factor with unknown cardiac involvement. It has been hypothesized that *ZFHX3* may be a regulatory factor for the JAK/STAT signaling cascade and this cascade may be involved in atrial fibrillation susceptibility; however, further study is needed to clarify this relationship [44], [45].

Fine mapping in African Americans has been proposed and employed to further refine signals from GWAS in European-descent populations with the premise that the lower levels of LD in the former population will be more likely to reveal the true casual SNP in a region of high LD observed in the latter population [46]. Also, the differences in allelic architecture known for these ancestral populations may reveal additional risk SNPs monomorphic or rare in one population but common in another. In this study, we attempted to capitalize on the lower levels of LD in African Americans compared with European Americans by densely genotyping independent SNPs for these three AF-associated regions. Previous studies of breast cancer and lipid traits have already capitalized on the differences in LD patterns, and, like this study, have identified new index SNPs in African Americans [47], [48]. Future studies incorporating new variation data from the 1000 Genomes Project [49] promise to identify the full spectrum of “risk” variants for AF and other traits in these cross-population fine-mapping experiments.

Unlike a previous report in the literature [30], European ancestry was not associated with increased AF risk in our African American cohort. Marcus et. al. genotyped 1,747 ancestry informative markers across the genome from the Illumina custom ITMAT-Broad-CARe array, while our study focused on 148 SNPs in three genomic regions previously identified in GWAS. The conflicting results may be due to the different study designs, such as the choice of markers (AIMs versus region specific). Also, the study by Marcus et. al. was larger in sample size compared with the present study.

The primary limitation of this study is the small sample size, which is a reflection of the observed reduced prevalence of AF among African Americans compared with European Americans. Assuming the European genetic effect size and the African American MAF, we were powered (>80%) to replicate two of the three index SNPs from prior GWA studies (rs13376333 and rs2200733). For the 145 non-index SNPs, we were underpowered to detect an effect size of <2.0 to 3.3 (dependent on MAF) at a liberal p-value threshold of 0.05. The power calculations presented here should be interpreted with caution given that the European genetic effect size was assumed to be equal to the African American effect size for the same variant, the latter of which there are no available data.

The limited sample size of the present study is also represented in the confidence intervals, which are wide. *ZFX3* rs16971547, for example, was associated with AF with an odds ratio of 8.162 but with an upper bound 95% confidence interval of 45.420 (p = 0.016). This variant is monomorphic in HapMap CEU but common in YRI (6%) and ASW (9%). Because of the limited case count and the low minor allele frequency (5%), the association identified between rs16971547 and AF in African Americans would benefit from further study.

Overall, the p-values presented here and in supplementary material were not corrected for multiple testing. Correction for multiple testing is complicated given that a fraction of the SNPs represented replication (that is, the index SNPs) while the others represented novel tests of association. Also, several methods for correction are available, each with limitations. For example, the Bonferroni correction is the most widely implemented method but it is often criticized for being overly conservative for datasets with even low levels of linkage disequilibrium between SNPs. Other methods of correction include the false discovery rate (FDR) and permutation testing [50]. None of the tests of association reported here survive correction for multiple testing when either Bonferroni or FDR are applied (data not shown). However, all SNPs except rs3866823 with an uncorrected p-value<0.05 have an emphirical p-value<0.05 after permutation testing. Replication of the associations reported here in an independent dataset is warranted given the small sample sizes and variability observed when implementing various corrections for multiple testing.

In conclusion, despite the overall limited power our study strongly suggests that the 1q21, 4q25, and 16q22 regions remain important in assessing AF risk among both European- and African-descent populations. These data also suggest the underlying biology identified by GWAS in European populations also applies to African American subjects.

## Supporting Information

Figure S1 **Locus Zoom plot for 1q21 region on chromosome 1.** Tests of association were performed for each SNP adjusted for age, body mass index, coronary artery disease, congestive heart failure, diabetes mellitus, and hypertension and are represented as circles or a diamond in the figure. SNPs are plotted based on chromosomal location (x-axis) and significance level (y-axis). Recombination rates are given on the opposing y-axis. The index association (rs484596) is denoted by the diamond. For this region, both the recombination rates, represented by the right axis, and linkage disequilibrium (based on HapMap phase II YRI), represented by the dot color in the SNP positions, are low.
(TIF)

Figure S2 **Locus Zoom plot for 4q25 region on chromosome 4.** Tests of association were performed for each SNP adjusted for age, body mass index, coronary artery disease, congestive heart failure, diabetes mellitus, and hypertension and are represented as circles or a diamond in the figure. SNPs are plotted based on chromosomal location (x-axis) and significance level (y-axis). Recombination rates are given on the opposing y-axis. The index association (rs4631108) is denoted by the diamond. For this region, both the recombination rates, represented by the right axis, and linkage disequilibrium (based on HapMap phase II YRI), represented by the dot color in the SNP positions, are low.
(TIF)

Figure S3 **Locus Zoom plot for 16q22 region on chromosome 16.** Tests of association were performed for each SNP adjusted for age, body mass index, coronary artery disease, congestive heart failure, diabetes mellitus, and hypertension and are represented as circles or a diamond in the figure. SNPs are plotted based on chromosomal location (x-axis) and significance level (y-axis). Recombination rates are given on the opposing y-axis. The index association (rs16971547) is denoted by the diamond. For this region, both the recombination.
(TIF)

Table S1 **Tests of association for atrial fibrillation in African Americans.** All tests of association are shown here regardless of significance, adjusted for age, body mass index, coronary artery disease, congestive heart failure, diabetes mellitus, and hypertension.
(DOC)

Table S2 **Comparison of allele frequencies between cases and controls and reference samples.** Coded allele frequencies for the ten most significant SNPs are shown for cases, controls, HapMap CEU, and HapMap YRI samples. Allelic frequency comparisons were performed with chi-square test and *F*-statistic (F_ST_).
(DOCX)
