# Supplementary figures and images for: Characterization of Genome-Wide Association-Identified Variants for Atrial Fibrillation in African Americans
Source: PLoS One. 2012 Feb 23;7(2):e32338. doi: 10.1371/journal.pone.0032338 (PMC3285683; doi:10.1371/journal.pone.0032338)

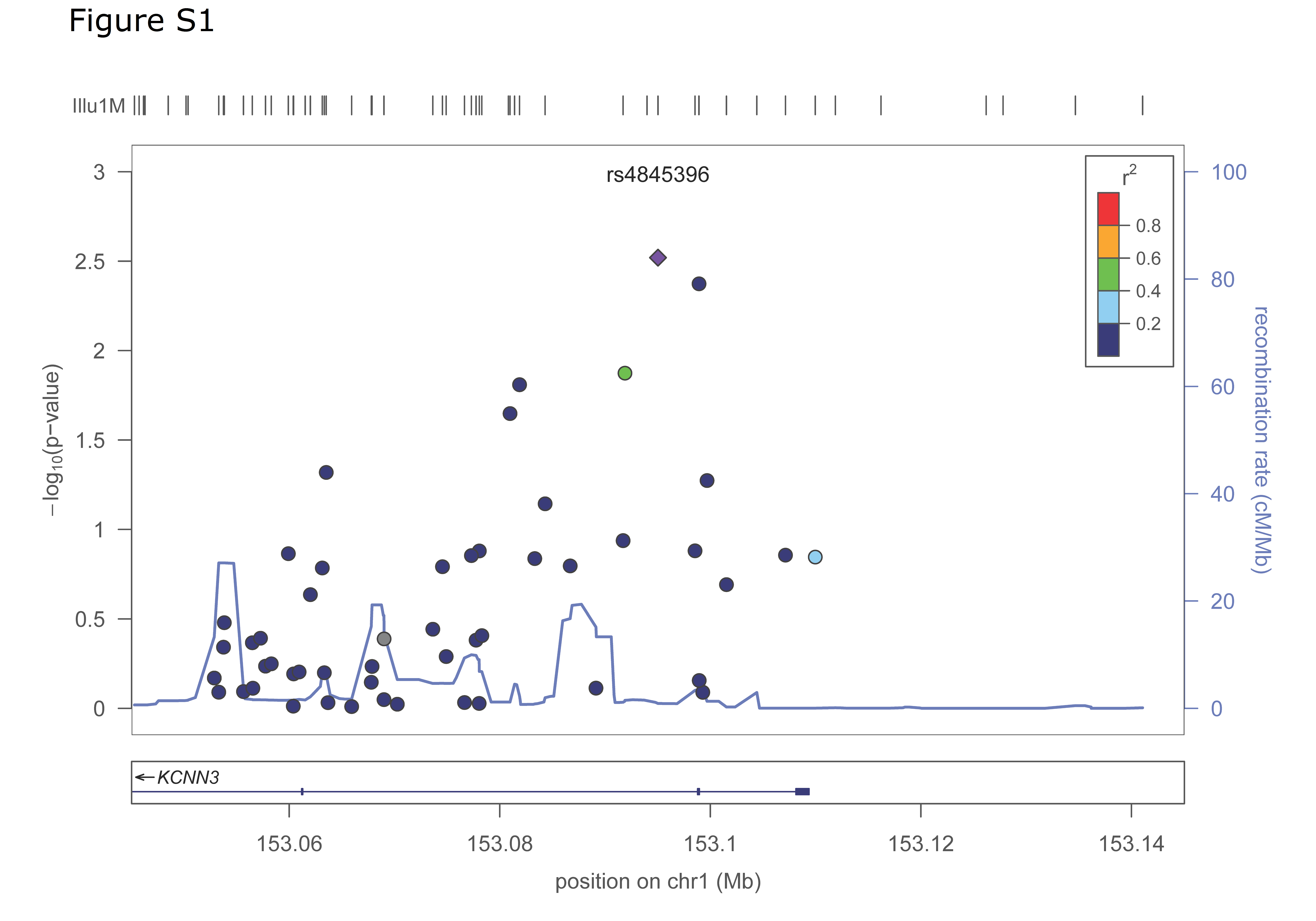

Supplement: Figure S1 — Locus Zoom plot for 1q21 region on chromosome 1. Tests of association were performed for each SNP adjusted for age, body mass index, coronary artery disease, congestive heart failure, diabetes mellitus, and hypertension and are represented as circles or a diamond in the figure. SNPs are plotted based on chromosomal location (x-axis) and significance level (y-axis). Recombination rates are given on the opposing y-axis. The index association (rs484596) is denoted by the diamond. For this region, both the recombination rates, represented by the right axis, and linkage disequilibrium (based on HapMap phase II YRI), represented by the dot color in the SNP positions, are low. (TIF) [file pone.0032338.s001.tif]

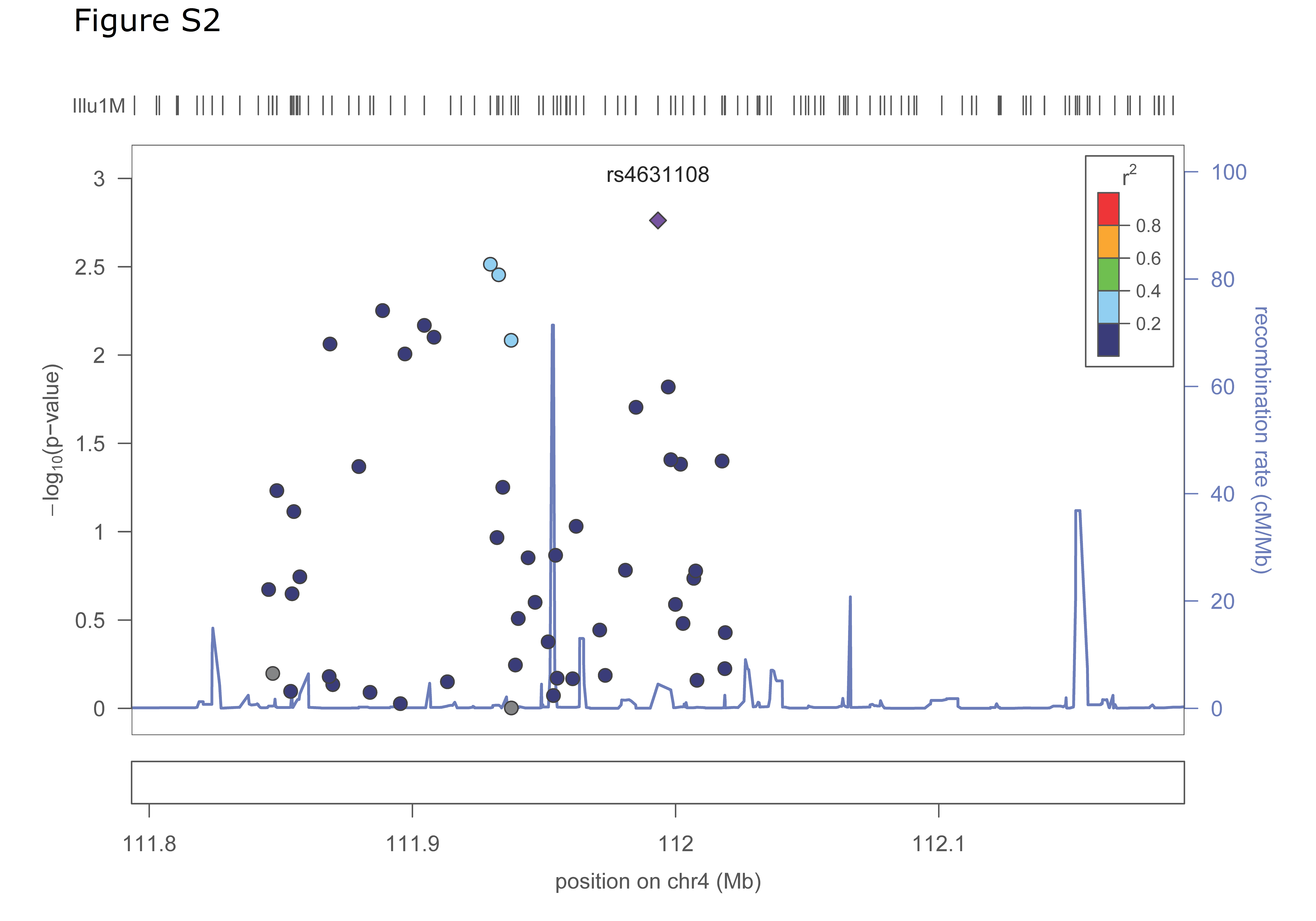

Supplement: Figure S2 — Locus Zoom plot for 4q25 region on chromosome 4. Tests of association were performed for each SNP adjusted for age, body mass index, coronary artery disease, congestive heart failure, diabetes mellitus, and hypertension and are represented as circles or a diamond in the figure. SNPs are plotted based on chromosomal location (x-axis) and significance level (y-axis). Recombination rates are given on the opposing y-axis. The index association (rs4631108) is denoted by the diamond. For this region, both the recombination rates, represented by the right axis, and linkage disequilibrium (based on HapMap phase II YRI), represented by the dot color in the SNP positions, are low. (TIF) [file pone.0032338.s002.tif]

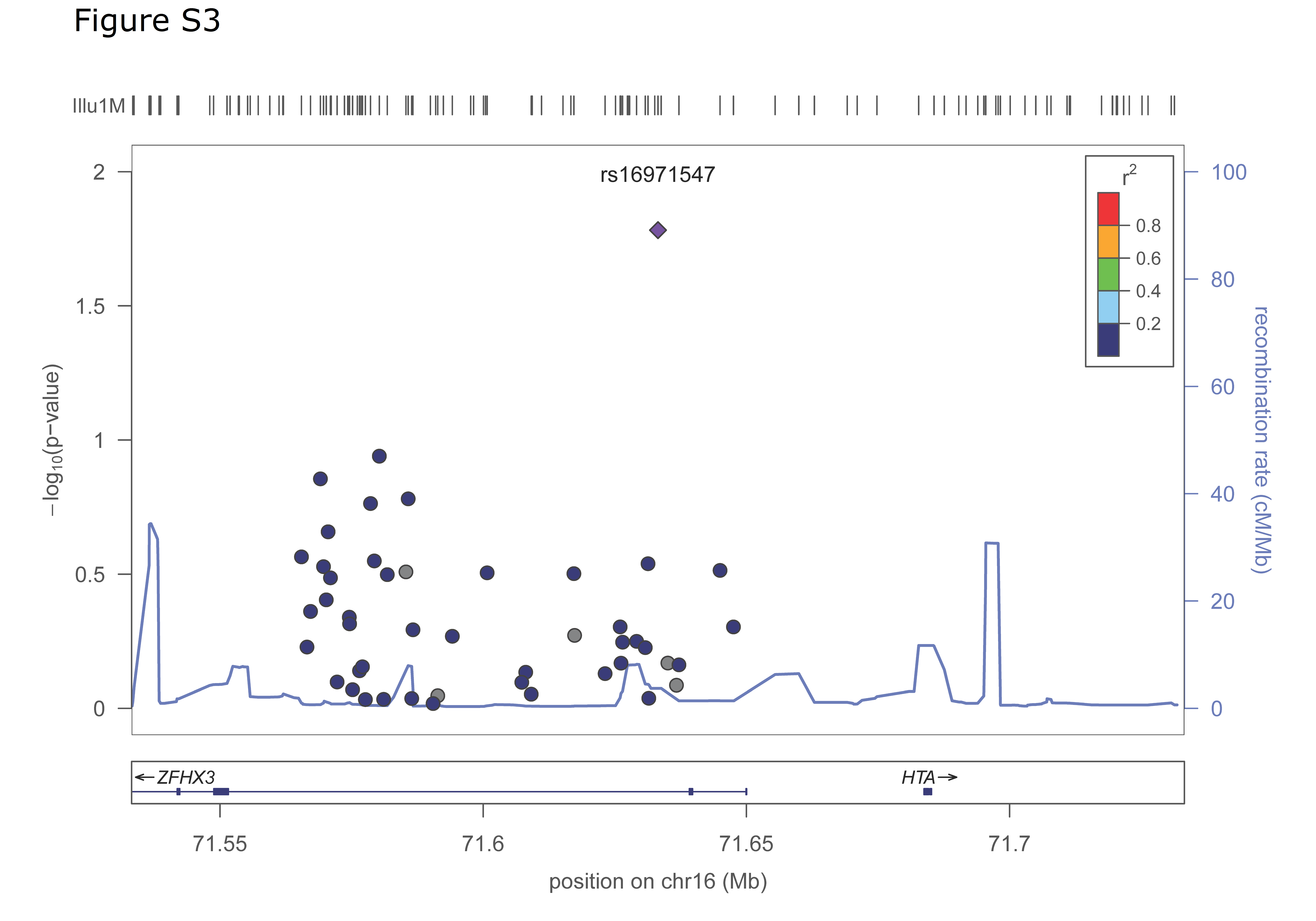

Supplement: Figure S3 — Locus Zoom plot for 16q22 region on chromosome 16. Tests of association were performed for each SNP adjusted for age, body mass index, coronary artery disease, congestive heart failure, diabetes mellitus, and hypertension and are represented as circles or a diamond in the figure. SNPs are plotted based on chromosomal location (x-axis) and significance level (y-axis). Recombination rates are given on the opposing y-axis. The index association (rs16971547) is denoted by the diamond. For this region, both the recombination. (TIF) [file pone.0032338.s003.tif]
