# Supplementary material for: Characterization of Genome-Wide Association-Identified Variants for Atrial Fibrillation in African Americans
Source: PLoS One. 2012 Feb 23;7(2):e32338. doi: 10.1371/journal.pone.0032338 (PMC3285683; doi:10.1371/journal.pone.0032338)
Supplement: Table S1 — Tests of association for atrial fibrillation in African Americans. All tests of association are shown here regardless of significance, adjusted for age, body mass index, coronary artery disease, congestive heart failure, diabetes mellitus, and hypertension. (DOC) [file pone.0032338.s004.doc]

**Table S1. Tests of association for atrial fibrillation in African Americans.** All tests of association are shown here regardless of significance, adjusted for age, body mass index, coronary artery disease, congestive heart failure, diabetes mellitus, and hypertension.

| **SNP** | **CHR** | **Chromosomal Location** | **Coded Allele** | **OR** | **95% Confidence Interval** | **P-value** |
| --- | --- | --- | --- | --- | --- | --- |
| rs4631108 | 4 | 111773867 | A | 3.430 | 1.587-7.417 | 0.002 |
| rs4845396 | 1 | 154828409 | A | 0.298 | 0.134-0.663 | 0.003 |
| rs2200733 | 4 | 111929618 | T | 3.283 | 1.495-7.207 | 0.003 |
| rs1906602 | 4 | 111713323 | C | 5.926 | 1.795-19.570 | 0.004 |
| rs4845397 | 1 | 154832304 | C | 0.321 | 0.147-0.699 | 0.004 |
| rs2634071 | 4 | 111669220 | A | 2.810 | 1.353-5.836 | 0.006 |
| rs4605724 | 4 | 111685081 | A | 4.723 | 1.535-14.530 | 0.007 |
| rs2723334 | 4 | 111688752 | G | 0.376 | 0.183-0.774 | 0.008 |
| rs6843082 | 4 | 111718067 | G | 2.629 | 1.284-5.383 | 0.008 |
| rs12647316 | 4 | 111649251 | T | 3.760 | 1.399-10.100 | 0.009 |
| rs10516563 | 4 | 111677722 | G | 3.283 | 1.332-8.097 | 0.010 |
| rs11264275 | 1 | 154825270 | G | 0.385 | 0.181-0.820 | 0.013 |
| rs4277843 | 4 | 111777749 | G | 2.280 | 1.173-4.434 | 0.015 |
| rs6426987 | 1 | 154815257 | C | 0.454 | 0.240-0.861 | 0.015 |
| rs16971547 | 16 | 73075708 | C | 8.162 | 1.467-45.420 | 0.017 |
| rs6838973 | 4 | 111765495 | T | 0.365 | 0.157-0.852 | 0.020 |
| rs13376333 | 1 | 154814353 | T | 2.292 | 1.124-4.672 | 0.022 |
| rs4285153 | 4 | 111778733 | A | 0.506 | 0.265-0.966 | 0.039 |
| rs11098092 | 4 | 111798201 | A | 2.143 | 1.037-4.432 | 0.040 |
| rs3866823 | 4 | 111782436 | T | 2.281 | 1.033-5.040 | 0.041 |
| rs11930528 | 4 | 111660194 | T | 2.103 | 1.025-4.315 | 0.043 |
| rs1984285 | 1 | 154796895 | G | 0.287 | 0.0834-0.988 | 0.048 |
| rs1218584 | 1 | 154833067 | C | 2.537 | 0.988-6.517 | 0.053 |
| rs2220427 | 4 | 111714889 | T | 2.378 | 0.978-5.782 | 0.056 |
| rs16997168 | 4 | 111629039 | T | 2.273 | 0.971-5.317 | 0.058 |
| rs1218565 | 1 | 154817687 | A | 0.526 | 0.262-1.058 | 0.072 |
| rs2723317 | 4 | 111635500 | A | 0.363 | 0.119-1.115 | 0.077 |
| rs11938968 | 4 | 111742752 | A | 0.501 | 0.223-1.122 | 0.093 |
| rs1906599 | 4 | 111712686 | T | 1.697 | 0.891-3.231 | 0.108 |
| rs4788686 | 16 | 73022767 | T | 0.274 | 0.054-1.369 | 0.114 |
| rs12058931 | 1 | 154825093 | A | 3.308 | 0.747-14.650 | 0.115 |
| rs1218587 | 1 | 154831925 | G | 1.654 | 0.860-3.179 | 0.131 |
| rs12128882 | 1 | 154811435 | C | 1.696 | 0.854-3.371 | 0.132 |
| rs17042215 | 4 | 111734958 | C | 2.415 | 0.759-7.691 | 0.1356 |
| rs4845673 | 1 | 154793296 | C | 0.418 | 0.133-1.317 | 0.136 |
| rs7531728 | 1 | 154840516 | A | 0.538 | 0.237-1.222 | 0.139 |
| rs9934475 | 16 | 73011577 | A | 0.594 | 0.299-1.184 | 0.139 |
| rs1218577 | 1 | 154810683 | G | 1.670 | 0.846-3.299 | 0.140 |
| rs723363 | 4 | 111724501 | G | 0.552 | 0.251-1.216 | 0.140 |
| rs2335407 | 1 | 154843347 | T | 0.583 | 0.283-1.199 | 0.142 |
| rs13375433 | 1 | 154816702 | G | 2.168 | 0.766-6.142 | 0.145 |
| rs1218559 | 1 | 154820067 | T | 0.506 | 0.196-1.308 | 0.160 |
| rs11584630 | 1 | 154807935 | A | 1.656 | 0.818-3.351 | 0.161 |
| rs11264268 | 1 | 154796520 | A | 0.608 | 0.301-1.225 | 0.164 |
| rs3853445 | 4 | 111761487 | C | 0.479 | 0.170-1.352 | 0.164 |
| rs2068360 | 16 | 73028249 | T | 0.573 | 0.261-1.258 | 0.165 |
| rs509952 | 4 | 111788170 | T | 0.611 | 0.304-1.228 | 0.166 |
| rs6416746 | 16 | 73021094 | T | 2.236 | 0.704-7.102 | 0.172 |
| rs2595087 | 4 | 111637776 | C | 0.409 | 0.111-1.509 | 0.180 |
| rs4833470 | 4 | 111787455 | G | 0.633 | 0.323-1.241 | 0.183 |
| rs6699080 | 1 | 154834911 | G | 1.534 | 0.794-2.964 | 0.203 |
| rs969642 | 4 | 111625925 | T | 1.675 | 0.745-3.764 | 0.212 |
| rs16971456 | 16 | 73013036 | G | 1.949 | 0.672-5.658 | 0.220 |
| rs10222783 | 4 | 111634826 | T | 0.649 | 0.324-1.302 | 0.224 |
| rs1218598 | 1 | 154795389 | C | 1.799 | 0.688-4.706 | 0.231 |
| rs3855819 | 4 | 111727163 | C | 0.697 | 0.376-1.290 | 0.251 |
| rs10213323 | 4 | 111780482 | T | 0.647 | 0.304-1.375 | 0.258 |
| rs16971447 | 16 | 73007970 | C | 1.899 | 0.604-5.964 | 0.272 |
| rs9927735 | 16 | 73021812 | T | 1.923 | 0.584-6.321 | 0.282 |
| rs9940321 | 16 | 73073808 | G | 0.690 | 0.347-1.370 | 0.289 |
| rs11075954 | 16 | 73012164 | T | 0.688 | 0.342-1.387 | 0.296 |
| rs4788697 | 16 | 73087494 | G | 0.656 | 0.293-1.470 | 0.306 |
| rs10033464 | 4 | 111720761 | T | 1.608 | 0.644-4.017 | 0.309 |
| rs16971481 | 16 | 73027823 | A | 1.782 | 0.584-5.434 | 0.310 |
| rs8060855 | 16 | 73043247 | A | 2.204 | 0.476-10.200 | 0.312 |
| rs1548374 | 16 | 73059729 | T | 0.674 | 0.313-1.453 | 0.314 |
| rs1858800 | 16 | 73024276 | A | 0.669 | 0.304-1.470 | 0.317 |
| rs9930445 | 16 | 73013482 | C | 1.378 | 0.727-2.611 | 0.326 |
| rs4033103 | 4 | 111783363 | G | 0.730 | 0.388-1.374 | 0.330 |
| rs1106235 | 1 | 154787213 | T | 0.580 | 0.193-1.740 | 0.331 |
| rs4032971 | 4 | 111751723 | G | 0.725 | 0.365-1.441 | 0.359 |
| rs1218547 | 1 | 154807018 | T | 1.340 | 0.716-2.506 | 0.360 |
| rs6843250 | 4 | 111799417 | G | 0.736 | 0.376-1.441 | 0.372 |
| rs1218574 | 1 | 154811677 | T | 0.741 | 0.373-1.470 | 0.391 |
| rs2040508 | 16 | 73012685 | C | 1.428 | 0.630-3.239 | 0.394 |
| rs7546937 | 1 | 154790651 | T | 1.680 | 0.496-5.688 | 0.404 |
| rs11264271 | 1 | 154802392 | C | 0.605 | 0.184-1.990 | 0.408 |
| rs11264274 | 1 | 154811127 | T | 1.534 | 0.548-4.292 | 0.414 |
| rs3866831 | 4 | 111732114 | T | 0.671 | 0.254-1.766 | 0.419 |
| rs1218603 | 1 | 154789875 | T | 1.310 | 0.671-2.555 | 0.429 |
| rs10852516 | 16 | 73009698 | C | 1.400 | 0.602-3.258 | 0.434 |
| rs1106236 | 1 | 154787136 | G | 0.734 | 0.327-1.650 | 0.454 |
| rs16971465 | 16 | 73017061 | G | 0.732 | 0.322-1.664 | 0.457 |
| rs4788489 | 16 | 73017118 | G | 0.791 | 0.410-1.525 | 0.483 |
| rs12373097 | 16 | 73068515 | C | 0.752 | 0.330-1.710 | 0.496 |
| rs8048267 | 16 | 73090039 | G | 0.782 | 0.384-1.589 | 0.496 |
| rs7193343 | 16 | 73029160 | T | 1.318 | 0.581-2.988 | 0.509 |
| rs4845394 | 1 | 154808287 | A | 0.791 | 0.393-1.594 | 0.512 |
| rs1548373 | 16 | 73059861 | T | 1.220 | 0.653-2.279 | 0.534 |
| rs8056528 | 16 | 73036633 | C | 1.225 | 0.642-2.338 | 0.538 |
| rs8053389 | 16 | 73071637 | T | 1.330 | 0.507-3.487 | 0.562 |
| rs1218601 | 1 | 154791676 | T | 1.408 | 0.442-4.484 | 0.563 |
| rs8057081 | 16 | 73068977 | T | 1.216 | 0.623-2.374 | 0.566 |
| rs11931959 | 4 | 111719685 | A | 0.827 | 0.432-1.583 | 0.567 |
| rs10752609 | 1 | 154791128 | G | 1.253 | 0.564-2.784 | 0.580 |
| rs1218550 | 1 | 154801247 | G | 0.790 | 0.342-1.827 | 0.582 |
| rs7199343 | 16 | 73009024 | T | 1.290 | 0.511-3.255 | 0.590 |
| rs8060701 | 16 | 73073289 | A | 1.216 | 0.593-2.492 | 0.593 |
| rs6817202 | 4 | 111799254 | T | 0.823 | 0.402-1.685 | 0.594 |
| rs9970364 | 1 | 154794318 | C | 1.241 | 0.522-2.955 | 0.625 |
| rs6681392 | 1 | 154796712 | C | 1.168 | 0.619-2.206 | 0.631 |
| rs2723313 | 4 | 111627489 | A | 1.178 | 0.601-2.306 | 0.633 |
| rs951241 | 1 | 154793798 | A | 1.195 | 0.566-2.524 | 0.641 |
| rs3866838 | 4 | 111753815 | A | 1.175 | 0.586-2.359 | 0.649 |
| rs7693227 | 4 | 111648928 | T | 0.656 | 0.101-4.277 | 0.659 |
| rs880309 | 4 | 111735539 | A | 0.879 | 0.482-1.604 | 0.674 |
| rs11804928 | 1 | 154786262 | T | 1.232 | 0.464-3.268 | 0.676 |
| rs728646 | 16 | 73077574 | A | 1.226 | 0.471-3.193 | 0.677 |
| rs879324 | 16 | 73068678 | T | 1.211 | 0.491-2.994 | 0.678 |
| rs7674295 | 4 | 111741438 | A | 0.886 | 0.499-1.573 | 0.679 |
| rs7204751 | 16 | 73079683 | T | 0.880 | 0.471-1.642 | 0.688 |
| rs521511 | 4 | 111788675 | T | 0.776 | 0.220-2.733 | 0.693 |
| rs1218586 | 1 | 154832322 | A | 1.267 | 0.384-4.186 | 0.698 |
| rs1858801 | 16 | 73019560 | A | 0.886 | 0.478-1.640 | 0.699 |
| rs17042150 | 4 | 111693848 | T | 1.327 | 0.304-5.780 | 0.706 |
| rs1218551 | 1 | 154801173 | G | 0.869 | 0.411-1.837 | 0.714 |
| rs16971474 | 16 | 73019004 | A | 0.856 | 0.363-2.022 | 0.723 |
| rs6836206 | 4 | 111650298 | C | 1.217 | 0.396-3.737 | 0.732 |
| rs11860416 | 16 | 73050576 | G | 1.285 | 0.305-5.403 | 0.733 |
| rs4788692 | 16 | 73065656 | C | 0.888 | 0.440-1.795 | 0.742 |
| rs906597 | 1 | 154822517 | C | 1.107 | 0.563-2.176 | 0.769 |
| rs12064718 | 1 | 154789924 | G | 0.901 | 0.447-1.816 | 0.770 |
| rs11640395 | 16 | 73014746 | T | 1.103 | 0.523-2.330 | 0.797 |
| rs4788689 | 16 | 73049830 | G | 0.909 | 0.434-1.901 | 0.799 |
| rs2723316 | 4 | 111634287 | T | 1.105 | 0.511-2.389 | 0.800 |
| rs10796933 | 1 | 154789036 | C | 1.117 | 0.466-2.679 | 0.804 |
| rs10027473 | 4 | 111664452 | G | 1.302 | 0.151-11.200 | 0.810 |
| rs2335406 | 1 | 154786686 | T | 0.906 | 0.404-2.029 | 0.811 |
| rs1218585 | 1 | 154832661 | A | 0.863 | 0.260-2.899 | 0.8116 |
| rs11641701 | 16 | 73079212 | A | 0.910 | 0.404-2.047 | 0.819 |
| rs3853444 | 4 | 111734136 | G | 1.110 | 0.393-3.136 | 0.844 |
| rs16971471 | 16 | 73017684 | A | 1.069 | 0.533-2.142 | 0.851 |
| rs2106261 | 16 | 73051620 | A | 1.048 | 0.559-1.964 | 0.884 |
| rs11264270 | 1 | 154802379 | G | 1.080 | 0.357-3.264 | 0.892 |
| rs11075958 | 16 | 73033869 | T | 0.909 | 0.216-3.818 | 0.896 |
| rs9940430 | 16 | 73073953 | T | 0.953 | 0.389-2.334 | 0.916 |
| rs756720 | 16 | 73028921 | A | 0.957 | 0.417-2.196 | 0.918 |
| rs2040507 | 16 | 73023587 | G | 1.043 | 0.436-2.494 | 0.924 |
| rs1218578 | 1 | 154810030 | C | 0.969 | 0.496-1.890 | 0.925 |
| rs11640106 | 16 | 73020116 | G | 1.035 | 0.507-2.110 | 0.926 |
| rs6681725 | 1 | 154797062 | G | 1.031 | 0.537-1.978 | 0.927 |
| rs12139030 | 1 | 154811420 | T | 1.044 | 0.364-2.997 | 0.936 |
| rs2634075 | 4 | 111675978 | A | 1.047 | 0.326-3.365 | 0.938 |
| rs1218549 | 1 | 154803645 | T | 0.979 | 0.529-1.811 | 0.946 |
| rs16971492 | 16 | 73032979 | A | 1.026 | 0.394-2.668 | 0.958 |
| rs951240 | 1 | 154793763 | C | 0.977 | 0.286-3.334 | 0.970 |
| rs1218552 | 1 | 154799310 | G | 0.988 | 0.461-2.116 | 0.975 |
| rs13105878 | 4 | 111718147 | A | 1.008 | 0.180-5.653 | 0.993 |
