# Supplementary material for: Characterization of Genome-Wide Association-Identified Variants for Atrial Fibrillation in African Americans
Source: PLoS One. 2012 Feb 23;7(2):e32338. doi: 10.1371/journal.pone.0032338 (PMC3285683; doi:10.1371/journal.pone.0032338)
Supplement: Table S2 — Comparison of allele frequencies between cases and controls and reference samples. Coded allele frequencies for the ten most significant SNPs are shown for cases, controls, HapMap CEU, and HapMap YRI samples. Allelic frequency comparisons were performed with chi-square test and F-statistic (FST). (DOCX) [file pone.0032338.s005.docx]

**Table S2 . Comparison of allele frequencies between cases and controls and reference samples.** Coded allele frequencies for the ten most significant SNPs are shown for cases, controls, HapMap CEU, and HapMap YRI samples. Allelic frequency comparisons were performed with chi-square test and *F*-statistic (F_ST_).

|  |  | **Coded Allele Frequencies** | | | | | **Coded Allele frequency comparisons**  **(P-value)** | | | | **F_ST_** | |
| --- | --- | --- | --- | --- | --- | --- | --- | --- | --- | --- | --- | --- |
| **SNP** | **Coded Allele** | Total | Cases | Controls | CEU | YRI | CEU vs.  Case | YRI vs. Case | CEU vs. Control | YRI vs. Control | CEU vs.Cases & Controls | CEU vs. Case |
| rs4631108 | A | 0.41 | 0.50 | 0.31 | 0.38 | 0.34 | 0.027 | 0.004 | 0.148 | 0.451 | <0.001 | 0.010 |
| rs4845396 | A | 0.28 | 0.21 | 0.35 | 0.48 | 0.13 | <0.001 | 0.059 | 0.018 | <0.001 | 0.040 | 0.070 |
| rs2200733 | T | 0.50 | 0.36 | 0.15 | 0.12 | 0.22 | <0.001 | 0.001 | 0.188 | 0.085 | 0.170 | 0.080 |
| rs1906602 | C | 0.14 | 0.19 | 0.09 | 0 | 0.20 | <0.001 | 0.706 | <0.001 | 0.003 | 0.090 | 0.120 |
| rs4845397 | C | 0.46 | 0.41 | 0.52 | 0.72 | 0.37 | <0.001 | 0.401 | <0.001 | 0.003 | 0.070 | 0.090 |
| rs2634071 | A | 0.43 | 0.53 | 0.34 | 0.20 | 0.55 | <0.001 | 0.679 | 0.001 | <0.001 | 0.060 | 0.120 |
| rs4605724 | A | 0.14 | 0.20 | 0.08 | 0.12 | 0.08 | 0.020 | 0.001 | 0.274 | 0.934 | <0.001 | 0.009 |
| rs2723334 | G | 0.50 | 0.40 | 0.61 | 0.20 | 0.60 | <0.001 | 0.879 | 0.001 | <0.001 | 0.080 | 0.140 |
| rs6843082 | G | 0.34 | 0.42 | 0.24 | 0.23 | 0.34 | <0.001 | 0.108 | 0.958 | 0.030 | 0.010 | 0.040 |
| rs12647316 | T | 0.19 | 0.28 | 0.11 | 0.12 | 0.12 | <0.001 | <0.001 | 0.777 | 0.777 | 0.007 | 0.040 |
